# Complete Genome Sequences of Two Corynebacterium macginleyi Strains Isolated from Infectious Keratitis

**DOI:** 10.1128/MRA.00207-21

**Published:** 2021-04-29

**Authors:** Susanna Sagerfors, Anja Poehlein, Bo Söderquist, Holger Brüggemann

**Affiliations:** a Department of Ophthalmology, Faculty of Medicine and Health, Örebro University, Örebro, Sweden; b Department of Genomic and Applied Microbiology, Institute of Microbiology and Genetics, University of Göttingen, Göttingen, Germany; c Department of Laboratory Medicine, Clinical Microbiology, Faculty of Medicine and Health, Örebro University, Örebro, Sweden; d Department of Biomedicine, Aarhus University, Aarhus, Denmark; University of Maryland School of Medicine

## Abstract

Corynebacterium macginleyi is a slow-growing, lipid-requiring bacterium that may cause ocular infections. Here, we report the complete genome sequences of two strains, T160811 and T180208, isolated from infectious keratitis. The two genomes consist of circular chromosomes of 2,431,961 bp and 2,481,998 bp, respectively, and contain high numbers of repetitive elements.

## ANNOUNCEMENT

Corynebacterium macginleyi was first described by Riegel et al. in 1995 (1). The Gram-positive, facultative anaerobic organism has been associated with a few cases of sepsis and infections related to foreign bodies (2, 3). However, the majority of studies on *C. macginleyi* report ocular infections, such as conjunctivitis, keratitis, and endophthalmitis (4–7). In 2000, Joussen et al. proposed that *C. macginleyi* is a conjunctiva-specific pathogen (4).

Here, we present the complete genome sequences of two *C. macginleyi* strains that were isolated from two patients with infectious keratitis in Örebro, Sweden (8). For primary isolation, corneal samples were directly inoculated onto gonococcal (GC) agar (GC medium base [Becton, Dickinson, Sparks, MD, USA] supplemented with 1% BBL IsoVitaleX enrichment) and incubated in 5% CO_2_ at 36°C for 5 days. Species identification was conducted by matrix-assisted laser desorption ionization–time of flight (MALDI-TOF) mass spectrometry as described previously (8). The same agar medium and growth conditions were used for subcultivation of the strains for DNA isolation. High-molecular-weight DNA (HWD) was isolated with the MasterPure complete DNA and RNA purification kit (Biozym, Hessisch Oldendorf, Germany). The quality of the isolated DNA was validated on an Agilent Bioanalyzer 2100 using an Agilent DNA 12000 kit (Waldbronn, Germany). The concentration and purity of the isolated DNA were determined using the Qubit double-stranded DNA (dsDNA) high-sensitivity (HS) assay kit (Life Technologies GmbH, Darmstadt, Germany). Illumina shotgun libraries were prepared using the Nextera XT DNA sample preparation kit and subsequently sequenced on a MiSeq system with the reagent kit v3 with 600 cycles (Illumina, San Diego, CA, USA). The reads were quality filtered using Trimmomatic v0.39 (9), resulting in 1,822,352 (T160811) and 1,597,180 (T180208) paired-end reads. For Nanopore sequencing, 1.5 μg unsheared HWD was used for the library preparation using the ligation sequencing kit 1D (SQK-LSK109) and the native barcode expansion kit (EXP-NBD103). Sequencing was performed for 72 h on a MinION Mk1B device with a SpotON R9.4.1 flow cell, using MinKNOW v19.06.8 and Guppy v3.2.1 for base calling (Oxford Nanopore, Oxford, UK). This resulted in 375,442 (T160811) and 28,770 (T180208) reads with *N*_50_ values of 5,016 bp and 4,911 bp, respectively. Unicycler v0.4.6 (10) was used to perform the hybrid assembly, resulting in one circular replicon per strain. The coverage was determined using QualiMap v2.2.1 (11) by mapping the Illumina and Nanopore reads onto the genome sequences using Bowtie 2 v2.3.5.1 (12) and minimap2 (13), respectively. Default parameters were used for all software unless otherwise specified. The closed chromosomes have an Illumina coverage of 190-fold (T160811) and 162-fold (T180208) and a Nanopore coverage of 273-fold and 162-fold, respectively; their sizes are 2,431,961 bp and 2,481,998 bp, respectively, and the GC content is 57.0% for both genomes.

Genome annotation, done with PGAP v5.1 (14), predicted 2,125 and 2,187 coding sequences (CDSs) in the chromosomes of strains T160811 and T180208, respectively. The annotation revealed that the genomes contain a high number of repetitive elements, with over 140 transposases, mainly of the IS*3* and IS*256* families. The closed genome sequences serve as references for future studies on ocular infections.

### Data availability.

The genome sequences of Corynebacterium macginleyi strains T160811 and T180208 have been deposited in GenBank under the accession no. CP068292 and CP068291, respectively. The raw reads have been deposited in the NCBI SRA database under the accession no. SRX10060782, SRX10060783, SRX10060784, and SRX10060785.

## References

[1] Riegel P, Ruimy R, de Briel D, Prévost G, Jehl F, Christen R, Monteil H. 1995. Genomic diversity and phylogenetic relationships among lipid-requiring diphtheroids from humans and characterization of *Corynebacterium macginleyi* sp. nov. Int J Syst Bacteriol 45:128–133. doi:10.1099/00207713-45-1-128. 7857793

[2] Villamil-Cajoto I, Rodríguez-Otero L, Villacián-Vicedo MJ, García-Zabarte MA, Aguilera-Guirao A, García-Riestra C, Regueiro BJ. 2008. Septicemia caused by *Corynebacterium macginleyi*: a rare form of extraocular infection. Int J Infect Dis 12:333–335. doi:10.1016/j.ijid.2007.07.004. 17981483

[3] Dobler G, Braveny I. 2003. Highly resistant *Corynebacterium macginleyi* as cause of intravenous catheter-related infection. Eur J Clin Microbiol Infect Dis 22:72–73. doi:10.1007/s10096-002-0870-6. 12582751

[4] Joussen AM, Funke G, Joussen F, Herbertz G. 2000. *Corynebacterium macginleyi*: a conjunctiva specific pathogen. Br J Ophthalmol 84:1420–1422. doi:10.1136/bjo.84.12.1420. 11090486 PMC1723327

[5] Ferreira CS, Figueira L, Moreira-Gonçalves N, Moreira R, Torrão L, Falcão-Reis F. 2018. Clinical and microbiological profile of bacterial microbial keratitis in a Portuguese tertiary referral center—where are we in 2015? Eye Contact Lens 44:15–20. doi:10.1097/ICL.0000000000000298. 27541969

[6] Suzuki T, Iihara H, Uno T, Hara Y, Ohkusu K, Hata H, Shudo M, Ohashi Y. 2007. Suture-related keratitis caused by *Corynebacterium macginleyi*. J Clin Microbiol 45:3833–3836. doi:10.1128/JCM.01212-07. 17913935 PMC2168495

[7] Ferrer C, Ruiz-Moreno JM, Rodriguez A, Montero J, Alio JL. 2004. Postoperative *Corynebacterium macginleyi* endophthalmitis. J Cataract Refract Surg 30:2441–2444. doi:10.1016/j.jcrs.2004.04.056. 15519105

[8] Sagerfors S, Poehlein A, Afshar M, Lindblad BE, Brüggemann H, Söderquist B. 2021. Clinical and genomic features of *Corynebacterium macginleyi*-associated infectious keratitis. Sci Rep 11:6015. doi:10.1038/s41598-021-85336-w. 33727638 PMC7966771

[9] Bolger AM, Lohse M, Usadel B. 2014. Trimmomatic: a flexible trimmer for Illumina sequence data. Bioinformatics 30:2114–2120. doi:10.1093/bioinformatics/btu170. 24695404 PMC4103590

[10] Wick RR, Judd LM, Gorrie CL, Holt KE. 2017. Unicycler: resolving bacterial genome assemblies from short and long sequencing reads. PLoS Comput Biol 13:e1005595. doi:10.1371/journal.pcbi.1005595. 28594827 PMC5481147

[11] García-Alcalde F, Okonechnikov K, Carbonell J, Cruz LM, Götz S, Tarazona S, Dopazo J, Meyer TF, Conesa A. 2012. QualiMap: evaluating next-generation sequencing alignment data. Bioinformatics 28:2678–2679. doi:10.1093/bioinformatics/bts503. 22914218

[12] Langmead B, Salzberg SL. 2012. Fast gapped-read alignment with Bowtie 2. Nat Methods 9:357–359. doi:10.1038/nmeth.1923. 22388286 PMC3322381

[13] Li H. 2018. minimap2: pairwise alignment for nucleotide sequences. Bioinformatics 34:3094–3100. doi:10.1093/bioinformatics/bty191. 29750242 PMC6137996

[14] Tatusova T, DiCuccio M, Badretdin A, Chetvernin V, Nawrocki EP, Zaslavsky L, Lomsadze A, Pruitt KD, Borodovsky M, Ostell J. 2016. NCBI Prokaryotic Genome Annotation Pipeline. Nucleic Acids Res 44:6614–6624. doi:10.1093/nar/gkw569. 27342282 PMC5001611

